# Supplementary material for: A Hyaluronic Acid Demilune Scaffold and Polypyrrole-Coated Fibers Carrying Embedded Human Neural Precursor Cells and Curcumin for Surface Capping of Spinal Cord Injuries
Source: Biomedicines. 2021 Dec 16;9(12):1928. doi: 10.3390/biomedicines9121928 (PMC8698735; doi:10.3390/biomedicines9121928)
Supplement: Supplementary file 1 [file biomedicines-09-01928-s001.zip › biomedicines-1466422-supplementary.pdf]

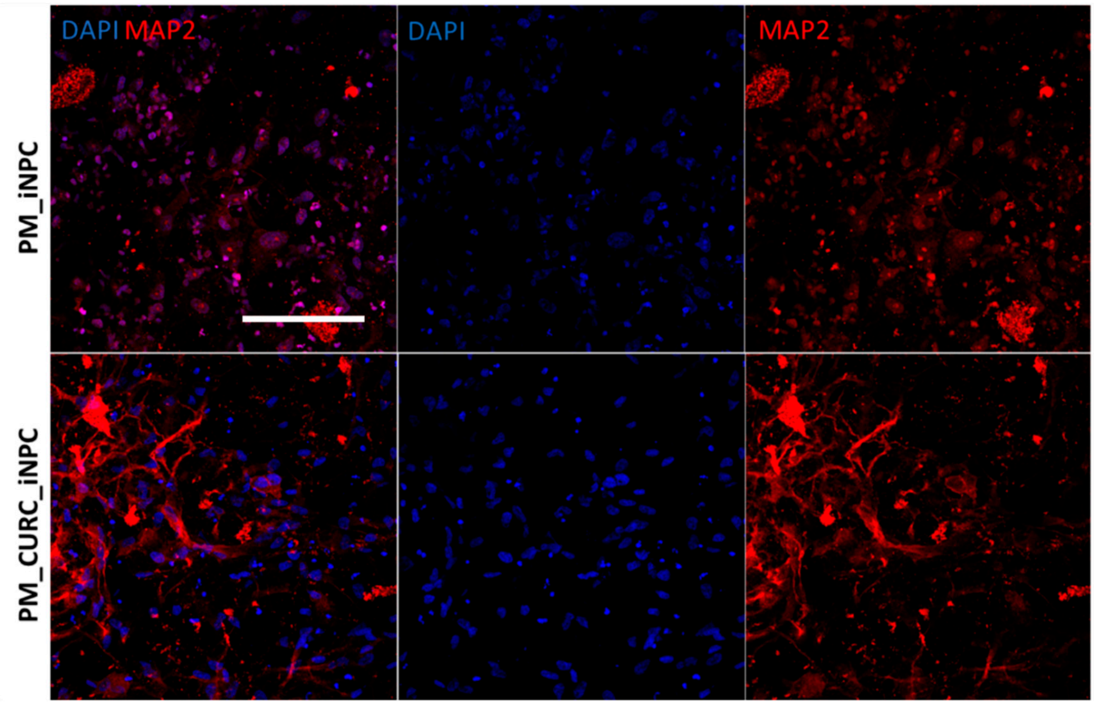

Figure S1. MAP2 positive staining for evaluation of neuronal maturation rates in iNPC-PM embedded with or without CURC. Confocal immunofluorescence images for MAP2 (RED) iNPC embedded into PM 0.15% (upper panels) or PM 0.15% mixed with CURC 5 µM (lower panels). Nuclei were stained with DAPI (blue). Scale bars: 100 µm.
